# New investigational drugs with single-agent activity in multiple myeloma

**DOI:** 10.1038/bcj.2016.53

**Published:** 2016-07-29

**Authors:** A M Rajan, S Kumar

**Affiliations:** 1 Department of Family Medicine, Medical College of Wisconsin, Milwaukee, WI, USA; 2 Division of Hematology, Mayo Clinic, Rochester, MN, USA

## Abstract

The treatment of multiple myeloma (MM) is rapidly evolving. In the United States, four drugs (panobinostat, ixazomib, daratumumab and elotuzumab) were approved for the treatment of MM in 2015. As a result of improved diagnosis and therapy, there has been a dramatic improvement in the outcome of MM in the last decade, probably more than any other malignancy. Numerous agents continue to be studied in preclinical models and in clinical trials, with many demonstrating clinical efficacy that appears promising enough to have a trajectory for regulatory approval. The purpose of this article is to summarize the current data and provide perspective on new investigational agents with promising single-agent activity in MM. The agents reviewed include Isatuximab, an anti-CD38 monoclonal antibody; marizomib, a new proteasome inhibitor; oprozomib, an oral proteasome inhibitor; filanesib (ARRY-520), a kinesin spindle protein inhibitor; dinaciclib, a cyclin-dependent kinase inhibitor; venetoclax (ABT-199), a selective BCL-2 inhibitor; and LGH-447, pan PIM kinase inhibitor.

## Introduction

Multiple myeloma (MM) is a clonal plasma cell malignancy that accounts for ~10% of hematologic malignancies.^1, 2^ It is a complex disease with several distinct cytogenetic subtypes, and is generally considered incurable in the majority of patients.^3, 4^ From the 1950s until the end of the 1990s, the mainstay of therapy of MM was alkylators (melphalan and cyclophosphamide), anthracyclines, corticosteroids (prednisone and dexamethasone)^5^ and in selected patients high-dose chemotherapy with autologous stem cell transplantation.^6, 7^ Subsequently, thalidomide,^8^ bortezomib^9^ and lenalidomide^10^ emerged as effective agents and greatly improved clinical outcome.^11, 12^ Thalidomide and lenalidomide are considered immunomodulatory drugs (IMiDs), although recent studies show that these drugs work by binding to and activating cereblon E3 ligase activity, resulting in the rapid ubiquitination and degradation of two specific B-cell transcription factors, Ikaros family zinc-finger proteins Ikaros (IKZF 1) and Aiolos (IKZF3). Bortezomib is a first-in-class proteasome inhibitor that acts by inhibiting the ubiquitin–proteasome catalytic pathway in cells by binding directly with the 20S proteasome complex. These three drugs have changed the treatment and outcome of MM dramatically, with many studies indicating at least a doubling of overall survival over the last decade.

In 2013, carfilzomib^13, 14, 15^ (a second-generation proteasome inhibitor) and pomalidomide^16^ (a newer more potent IMiD) were approved for clinical use based on clinical efficacy in phase 2 and 3 trials. More recently, in 2015, four other drugs were approved for MM, greatly expanding the therapeutic armamentarium. These include panobinostat^17^ (a pan-histone deacetylase inhibitor), ixazomib (an oral proteasome inhibitor), elotuzumab (a monoclonal antibody targeting SLAMF7) and daratumumab (a monoclonal antibody targeting CD38) and have been approved in the United States for the treatment of MM, substantially expanding the number of treatment regimens available for patients. There is no doubt that the arrival of several new drugs in the last 3 years will further increase outcomes for MM and related disorders. The drugs approved in the United States so far for the treatment of MM have been reviewed in detail in several original publications and reviews and are beyond the scope of this review.

## New investigational drugs

**Figure 1:**
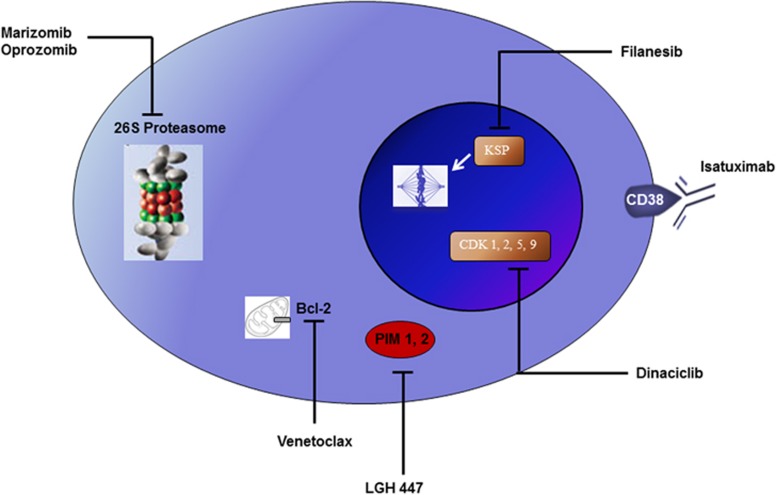
New active drugs in the treatment of multiple myeloma. Bcl-2, B-cell lymphoma 2; CDK, cyclin-dependant kinase; KSP, kinesin spindle protein; PIM, proto-oncogene serine/threonine-protein kinase.

A wide array of drugs is being developed for the treatment of MM, including some with unique mechanisms of action (Figure 1). Most of these drugs are in early stages of development, with efficacy data limited to preclinical models. However, many new drugs are already showing significant single-agent activity in MM in phase 1 and 2 clinical trials, and hence there is a high likelihood that they will be eventually approved for the treatment of the disease in the near future. The development of these agents for regulatory approval is proceeding in parallel with efforts to develop new active combinations for clinical use. The most promising investigational new agents with significant single-agent activity in MM include isatuximab, an anti-CD-38 monoclonal antibody; marizomib, a new proteasome inhibitor; oprozomib, an oral proteasome inhibitor related to carfilzomib; filanesib (ARRY-520), a kinesin spindle protein (KSP) inhibitor; dinaciclib, a cyclin-dependent kinase (CDK) inhibitor; venetoclax (ABT-199), a selective BCL-2 inhibitor; and LGH-447, pan PIM kinase inhibitor (Table 1).

## Isatuximab (formerly referred to as SAR650984*;* Sanofi, Paris, France)

### Mechanism of action

Isatuximab is a humanized IgG1 monoclonal antibody that binds to a specific epitope on the human CD38 receptor. CD38 is a type 2 transmembrane protein expressed on both hematopoietic and non-hematopoietic tissues, with the highest density being on plasma cells and germinal center B cells. Approximately 80–100% of all myeloma cells express high levels of CD38 protein on their surface, making it a very good therapeutic target.^18^ CD38 functions as a receptor involved in the transmission of activation and proliferation signals, as well as an ectoenzyme that has a role in calcium signaling and cell survival.^19^ CD38 receptor-mediated signaling produces a variety of downstream effects, and has variable signaling impact depending on several factors. It has a role in T-cell activation mediated by the downstream activation of nuclear factor kB. CD38 can also exist in a soluble form in the plasma.

Daratumumab, another CD38-targeting monoclonal antibody, has already shown significant clinical activity with a favorable toxicity profile in patients with relapsed refractory MM (RRMM). Isatuximab binds to a different epitope on CD38 and may have more potent inhibition of its ectozyme function than daratumumab, thereby having the potential for some non-cross-reactivity. Further, whereas daratumumab induces crosslinking-dependent apoptosis, isatuximab may be able to promote apoptosis even without crosslinking.^19^

Isatuximab may act through many different mechanisms including the following: (1) antibody-dependent cellular cytotoxicity; (2) complement-dependent cytotoxicity; (3) antibody-dependent cellular phagocytosis; (4) direct apoptosis; and (5) inhibition of myeloid-derived suppressor cells resulting in the release of T-cell suppression.^18^

### Clinical results in myeloma

Isatuximab has shown significant clinical activity in patient populations who were heavily pretreated with at least five lines of therapy, including pomalidomide, lenalidomide and/or carfilzomib.^18^

The first human phase 1/2 dose-escalation study by Martin *et al.* tested the single-agent activity of isatuximab in patients with CD38-positive RRMM.^20^ The maximum tolerated dose (MTD) was not reached at 20 mg kg^−1^ per week. The majority of patients in this trial had received at least six prior regimens of therapy. Infusion-related reactions were common and occurred primarily during the first infusion. A partial response rate of ~25% was observed at the 20-mg kg^−1^ once weekly dose regimen (Table 2). On the basis of these results an expansion cohort study of 105 additional patients are being treated with isatuximab at 20 mg kg^−1^ once weekly for 4 weeks, followed by a maintenance dose of 10 mg kg^−1^ for every 2 weeks. On the basis of these promising results, isatuximab will be moving to phase 3 testing soon.

### Side effects

The most common treatment-emergent adverse events (AEs) of isatuximab have been fatigue, nausea, cough and dyspnea. Hematologic treatment-emergent AEs include anemia (100%) thrombocytopenia (65%) and neutropenia (40%).^20^ Infusion-associated reactions occur in ~50% of patients and are limited mainly to cycle 1. They include chills, dyspnea, nausea, pyrexia, fatigue, flushing, chest pain and cough.

### Dosage schedules

The planned dosing schedule for isatuximab as a single agent is 20 mg kg^−1^ intravenously weekly × 4 weeks, followed by maintenance therapy of 10 mg kg^−1^ intravenously every 2 weeks.^20^ The optimal dose and dosing schedule in combination with other drugs is still to be determined in further studies.

### Ongoing trials

There are several studies that are currently ongoing evaluating isatuximab in combination with various other chemotherapy drugs for MM, including lenalidomide–dexamethasone, pomalidomide–dexamethasone, cyclophosphamide, bortezomib and dexamethasone, and carfilzomib. Preliminary results suggest that the toxicity profile is favorable with a combination of isatuximab and lenalidomide–dexamethasone. Phase 3 studies are being planned and are expected to open later this year. Isatuximab may also have a role in the treatment of other hematological malignancies, solid tumors and antibody-mediated autoimmune diseases based on its varied mechanisms of action.

### Anticipated Role in MM Treatment

Given that daratumumab has already been approved for clinical use in the United States for RRMM, the potential role of isatuximab is still unclear. As these are monoclonal antibodies with propensity for severe infusion-related reactions, it is possible that patients intolerant to one may be able to tolerate the other drug. It is also likely that the drugs may have some non-cross-reactivity as they bind to different epitopes. Cost is a major concern in myeloma therapy, and the availability of two monoclonal CD38 antibodies on the market may help with affordability.

## Marizomib (formerly referred to as NPI-0052, salinosporamide A; Triphase Research, Kasaba hobi, Mysore, Karnataka, India)

### Mechanism of action

Marizomib is a second-generation proteasome inhibitor that is structurally different from bortezomib and other proteasome inhibitors (Table 3). The proteasome is a large multicatalytic enzyme essential for the regulation of cell function and growth by controlling the orderly breakdown of intracellular proteins. (Rajkumar *et al.*^1^) By inhibiting the proteasome pathway marizomib leads to cell-cycle arrest and apoptosis of cancer cells.

Proteasome inhibitors have emerged as important drugs for the treatment of hematologic malignancies. In May 2003, the Food and Drug Administration approved the first-in-class proteasome inhibitor bortezomib (Velcade) for the treatment of RRMM. Since then bortezomib has become an essential component of myeloma therapy for all stages of the disease. Two other proteasome inhibitors, carfilzomib and ixazomib, have since been approved.

Marizomib is a second-generation beta-lactone-gamma-lactam proteasome inhibitor with the unique ability to effectively inhibit not just one but all three proteolytic activities of the proteasome. Its distinctive structure and pharmacologic activities enable it to act as a one-of-a-kind inhibitor of the 20S proteasome. The preclinical efficacy of marizomib was initially evaluated in both hematologic malignancies and solid tumors.^21^ In preclinical studies, marizomib had greater activity on MM cells compared with bortezomib,^22^ without significant toxicity on other hematopoietic cell lines or normal human neural stem cells. In fact, marizomib had single-agent activity against primary myeloma cells refractory to thalidomide-dexamethasone as well as bortezomib. In mouse myeloma models, marizomib reduced tumor recurrence with dosages as low as 0.15 mg kg^−1^ intravenously. It was also found to be synergistic with lenalidomide in these models, demonstrating enhanced ability to inhibit 20S proteasome proteolytic activity.

### Clinical results in myeloma

On the basis of these preclinical results, a Phase 1 single-arm trial in RRMM to determine the MTD of marizomib was undertaken. Marizomib was administered intravenously on two different schedules 0.025–0.7 mg m^−^^2^ once weekly on days 1, 8 and 15 of 4-week cycles (schedule A) and 0.15–0.6 mg m^−^^2^ twice weekly on days 1, 4, 8 and 11 of 3-week cycles (schedule B) with the addition of dexamethasone permitted in schedule B. All patients had RRMM and had received an average of five to seven prior treatment regimens. Thirty-two patients received schedule A, and 36 patients received schedule B. The trial concluded that 0.7 mg m^−^^2^ infused over 10 min was the optimal R2PD for schedule A, and 0.5 mg m^−^^2^ infused over 2 h for schedule B. Significant clinical activity was observed in both schedules. Minimal response or better was seen in 2 of 68 patients (3%), with 3 additional patients responding after the addition of dexamethasone (Table 2). The authors concluded that marizomib had activity in RRMM and recommended further testing and combination studies with other chemotherapeutic drugs.

In a separate study, Spencer *et al.*^23^ enrolled 14 patients into a dose-escalation trial of marizomib in combination with pomalidomide and dexamethasone. Patients had received multiple prior regimens (median 4.5, range 2–15), including both lenalidomide and bortezomib, and were refractory to their last therapy. Fifty percent had been previously treated with carfilzomib. Marizomib was administered intravenously at 0.3–0.5 mg m^−^^2^ over 2 h on days 1, 4, 8 and 11 in combination with pomalidomide and dexamethasone repeated every 28 days. A decrease in monoclonal (M) protein was seen in all patients by the end of their first treatment cycle. Of 11 evaluable patients, six (54%) achieved a partial response, two (12%) had minimal response and three (27%) achieved stable disease by the third cycle according to the International Myeloma Working Group criteria.

### Side effects

Common AEs include fatigue, nausea, vomiting, headache, dizziness and fever. However, unlike with bortezomib, peripheral neuropathy was not seen.^24^ DLTs include hallucinations and reversible cognitive changes. Thrombocytopenia and neutropenia were seen less frequently.

### Dosage schedule

The recommended dosing schedule for further studies is 0.5 mg m^−^^2^ intravenously over 2 h, given days 1, 4, 8 and 11 of a 21-day cycle. This schedule was recommended based on the observed activity with manageable toxicity.

### Ongoing trials

Currently, trials are ongoing, evaluating marizomib in combination with other drugs. A Phase 1 combination study of pomalidomide, marizomib and low-dose dexamethasone in RRMM is actively recruiting. Phase 2 and 3 studies are being planned.

### Anticipated role in myeloma

Marizomib has clearly shown activity as a single agent as well as in combination in patients with RRMM. However, its clinical role will depend on efficacy in patients who are refractory to or intolerant to other proteasome inhibitors. As it has minimal neurotoxicity, and appears to have activity in patients who are refractory to bortezomib, it is an agent that is worthy of further investigation. Further, the availability of multiple proteasome inhibitors will also enable us to tailor therapy according to the patients' needs, and provide competitive pressure on cost.

## Oprozomib (formerly referred to as ONX-0912; Amgen Inc., Thousand Oaks, CA, USA)

### Mechanism of action

Oprozomib is a new oral, irreversible proteasome inhibitor. Its mechanism of action is similar to marizomib, specifically acting via inhibition of chymotrypsin-like activity of the proteasome. It is a tripeptide epoxyketone that is structurally different from bortezomib. It can be best described as an oral analog of carfilzomib, a currently Food and Drug Administration-approved treatment for MM therapy. It exhibits similar properties through its anti-angiogenic and proapoptotic activity both *in vitro* and *in vivo* studies.^21^ Oprozomib results in activation of caspase-8, caspase-9, caspase-3 and poly(ADP) ribose polymerase, and inhibits the migration of MM cells. It induces apoptosis in MM cells resistant to bortezomib-based therapies. Oprozomib significantly reduced tumor progression and resulted in prolonged survival in animal models. Chauhan *et al.*^25^ demonstrated that oprozomib-treated mice had decreased tumor growth and angiogenesis. It also enhanced the antimyeloma activity of bortezomib, lenalidomide–dexamethasone and a pan-histone deacetylase inhibitor. Currently, the preclinical data suggest its efficacy to be on par with carfilzomib, and these results provide the rationale for clinical testing.

### Clinical results in myeloma

A Phase 1b/2 single-agent open-label study by Vij *et al.*^26^ was conducted in 106 patients, of whom 68 had MM that had relapsed after more than one prior therapy, to determine the MTD and safety profile of oprozomib. Oprozomib was administered once daily on days 1, 2, 8 and 9 of a 14-day cycle (schedule 1) or on days 1–5 of a 14-day cycle (schedule 2). The initial dose was 150 mg per day and escalated in 30 mg increments to a maximum of 330 mg per day. The phase 2 dose was determined to be 240 mg per day on days 1–5 of a 14-day cycle. A partial response rate of ~35% was seen in carfilzomib-naive patients in the phase 1b portion of the study; the approximate response rate in bortezomib-refractory patients was 14%. In the phase 2 portion of the study, the response rate (PR or better) was 27% in carfilzomib-refractory patients (*n=*11), 33% in carfilzomib-sensitive patients (*n=*12) and 25% in bortezomib-refractory patients (*n=*12; Table 2). The MTD of oprozomib was determined as 300 mg per day in the first schedule and 240 mg per day in the second schedule. Preliminary data suggest that step-up dosing is associated with improved tolerability and fewer adverse effects. Single-agent oprozomib has promising antitumor activity with target enrollment for the phase 2 portion set at 94 patients with MM to receive a new extended release formulation of oprozomib.^26^

In another phase 1b/2 study by Hari *et al.*,^27^ oprozomib was tested in combination with dexamethasone in a multicenter, single-arm phase 1b/2 study to evaluate the safety and tolerability in 29 patients with RRMM. Patients had received at least one to five prior regimens of therapy including at least one with lenalidomide and/or bortezomib. Oprozomib doses started at 210 mg per day and were administered on days 1, 2, 8 and 9 of a 14-day cycle (2/7 schedule) or on days 1–5 of a 14-day cycle (5/14 schedule) with escalation in 30 mg increments. Dexamethasone was given orally on days 1, 2, 8 and 9 of a 14-day cycle. The 14 patients on schedule 2/7 had no dose-limiting toxicities (DLTs); however, three DLTs were observed in the 15 patients on schedule 5/14 (grade 2 subarachnoid hemorrhage, grade 3 transaminitis and grade 4 thrombocytopenia). A partial response was achieved in 5 of the 12 evaluable patients (schedule 2/7) for an overall best response rate of 41.7%. No partial responses were seen among the seven evaluable patients on the 5/14 schedule. The authors concluded that the combination of oprozomib and dexamethasone had clinical activity warranting further study.^27^

A phase 1b/2 multicenter trial tested the efficacy of the combination of oprozomib, pomalidomide and dexamethasone in 31 patients with RRMM who failed at least two or more prior consecutive cycles of bortezomib and either lenalidomide or thalidomide therapy. Patients were administered oprozomib 150 mg per day orally once a day on days 1–5 and 15–19 (5/14 schedule, *n*=4) or at 210 mg per day on days 1, 2, 8, 9, 15, 16, 22 and 23 (2/7 schedule, *n=*17) of 28-day cycles with subsequent escalations. Pomalidomide 4 mg was given orally on days 1–21, and dexamethasone 20 mg orally on days 1, 2, 8, 9, 15, 16, 22 and 23. A partial response or better was observed in two out of four patients (50%) in the 5/14 schedule, and in 10 of 17 patients (59%) in the 2/7 schedule. The MTD of oprozomib was 210 mg per day based on the safety and efficacy data available in this study. This ongoing study suggests that there is significant activity and benefit from using the oprozomib, pomalidomide and dexamethasone combination that is well tolerated with minimal side effects.

### Side effects

In phase 1 studies the DLTs of oprozomib were hypotension, diarrhea and thrombocytopenia.^26^ The most common AEs of single agent oprozomib in patients with MM are anemia, thrombocytopenia, nausea, vomiting and diarrhea.^27^ It also appears to be well tolerated in combinations with pomalidomide and dexamethasone.^28^

### Dosage schedule

The recommended phase 2 dose of oprozomib is 240 mg per day once daily on days 1, 2, 8 and 9 of a 14-day cycle, stepped up by 30 mg increments as tolerated to a maximum dose of 300 mg per day. An alternate schedule is oprozomib 150 mg per day on days 1–5 of a 14-day cycle stepped up by 30 mg increments to a maximum dose of or 180 mg  per day.^26^

### Ongoing trials

Besides studies in RRMM, a phase 1b/2 clinical trial combining oprozomib with melphalan–prednisone is ongoing in patients with newly diagnosed MM who are ineligible for stem cell transplantation. Another phase 1/2 trial is evaluating to two novel combinations of oprozomib, namely oprozomib–cyclophosphamide–dexamethasone and oprozomib–lenalidomide–dexamethasone, in patients with newly diagnosed MM.

### Anticipated Role in MM

Given the known activity of proteasome inhibitors in MM, we expect oprozomib to have a major role in MM therapy. The primary advantage with oprozomib is the ease of administration via the oral route. As with marizomib, the availability of multiple proteasome inhibitors will enable us to select the best agent based on activity, toxicity and preferred route of administration for patients.

## Dinaciclib (formerly referred to as SCH727965s, MK-7965; Merck/Ligand Pharmaceuticals, Kenilworth, NJ, USA)

### Mechanism of action

There is significant upregulation of cyclin D1, D2 and D3 expression in a majority of patients with MM, providing rationale for the testing CDK inhibitors in this disease. When cyclin binds to CDK it forms a complex that becomes an active kinase and an integral mediator of the cell cycle involved in regulating transcription, mRNA processing and cellular differentiation. Dinaciclib is a potent, small molecule inhibitor of CDK that interacts with the acetyl-lysine recognition site of bromodomains.^29^ Dinaciclib specifically targets CDK1, CDK2, CDK 5 and CDK9.^30^ CDK1- and CDK5-dependent mechanisms correlate with cyclin-B and p35 complex activity and help to inhibit the unfolded protein response.^31^ *In vitro* studies show that inhibiting CDK-5 enhances the activity of proteasome inhibitors. It disrupts critical cell survival mechanisms such as the unfolded protein response in myeloma cells, thereby disabling the cells' ability to prevent damage caused by other anticancer drug therapies.

### Clinical results in myeloma

A Phase 1/2 single-agent open-label study by Kumar *et al.*,^30^ was conducted on 29 patients who had relapsed MM after receiving five or less prior lines of therapy (including lenalidomide, thalidomide or pomalidomide) to determine the MTD and safety profile of dinaciclib. Dinaciclib was administered on day 1 of a 21-day cycle at 30, 40 and 50 mg m^−^^2^ doses, and the MTD was determined to be 50 mg m^−^^2^. The overall response rate was highest (33%) in the six patients treated with the 40 mg m^−^^2^ dose. Of the 27 evaluable patients, three (11%) achieved partial response or better, including two with very good partial response; two other patients achieved minimal response for an overall response rate of 18.5% (Table 2). An additional 10 (37%) patients obtained some degree of M protein stabilization or decrease. The results indicate promising activity of dinaciclib in RRMM.

### Side effects

The most common hematologic AEs observed were leukopenia (27%) and thrombocytopenia (60%), followed by non-hematologic AEs of diarrhea (87%), fatigue (67%), nausea (53%), gastrointestinal symptoms and alopecia.^30^ Dinaciclib is well tolerated overall, and the primary side effects are gastrointestinal symptoms, which are mild to moderate.

### Dosing schedule

The current recommended dosing schedule for further studies is 50 mg m^−^^2^ intravenous infusion over 2 h on day 1 of a 21-day cycle for a maximum of 12 cycles. This schedule was recommended based on the observed activity in the phase 2 study described above.^30^

### Ongoing trials

There are various ongoing trials testing the efficacy of dinaciclib in other hematologic malignancies. Currently, a phase 1 trial is evaluating the MTD of dinaciclib and bortezomib, when used in combination with dexamethasone, in two different schedules, for treatment of relapsed MM. The study evaluates weekly and every 3-week dosing schedules for dinaciclib. The hypothesis is that the ability of dinaciclib to enhance the activity of proteasome inhibitors, such as bortezomib, will produce additive or synergistic effects.^31^

### Anticipated Role in MM

Dinaciclib has shown significant activity as a single agent in MM therapy thus far. Its role in the treatment of various other hematological malignancies and solid tumors is currently under investigation. It is a promising drug with a new mechanism of action based on its characteristic ability to regulate and inhibit various stages in the cell cycle. In addition to its ability to enhance the activity of drugs such as bortezomib, it may be capable of enhancing the activity of other drugs, and this could potentially generate several new combinations for myeloma. An effective well-tolerated drug such as dinaciclib can reduce exposure to cytotoxic chemotherapy and minimize side effects.

## Filanesib (formerly referred to as ARRY-520; Array BioPharma)

### Mechanism of action

Filanesib is one of the newest anticancer drugs, developed by Array BioPharma (Boulder, CO, USA), as a highly selective, targeted inhibitor of KSP.^32^ Kinesin spindle proteins are a part of a larger kinesin family of microtubule motor proteins that have a vital role in cell division.^33^ Its major function is to mediate the separation of centrosomes, and it also functions to provide bipolar spindle assembly and maintenance. Filanesib acts by inhibiting the normal function of these proteins and arrests the cell cycle in mitosis, resulting in monoastral microtubule arrays, leading inevitably to cell death. It has been found that in cells arrested by KSP inhibitors, an anti-apoptotic protein known as myeloid cell leukemia 1 (Mcl-1) is rapidly depleted resulting in cell death.^34^ Therefore, cells that are dependent on this pro-survival protein, Mcl-1, such as myeloma cells, are especially sensitive to filanesib.^35^ Filanesib is one of a several KSP inhibitors currently being studied in clinical trials as a novel anticancer chemotherapeutic drug. Preclinical studies suggest single-agent activity of filanesib in several *in vivo* models of MM alone and in combination with IMiDs.^36^

### Clinical results in myeloma

A Phase 2 trial evaluated filanesib alone (cohort 1) and in combination with low-dose dexamethasone (Cohort 2) in patients with RRMM treated with prior regimens including bortezomib and an immunomodulatory agent. Filanesib was administered in a 1.5-mg m^–^^2^ per day dose intravenously on days 1 and 2 every 2 weeks in (Cohort 1) and in a 1.5 mg m^–^^2^ per day intravenous dose with a 40-mg oral dose of low-dose dexamethasone weekly. Of the 32 patients enrolled in cohort 1 (median of six prior regimens, range 2–19), 5 (16%) achieved a partial response, with an additional patient achieving minimal response for an overall response rate of 19%. In patients with dual refractory disease to both bortezomib and lenalidomide, the overall response rate was 15%. In cohort 2 (median of 10 prior regimens, range 5–13) of the 18 evaluable patients, four (22%) achieved partial response, with one additional patient achieving minimal response for an overall response rate of 28%.^37^

A subsequent study of filanesib was conducted in combination with carfilzomib in patients with RRMM. Carfilzomib was administered intravenously at 27 mg m^−^^2^ (Cohort A) and escalated to 56 mg m^−^^2^ in combination with dexamethasone (Cohort B) on days 1, 2, 8, 9, 15 and 16 of a 28-day cycle. Filanesib 1.5 mg m^−^^2^ was administered intravenously on days 1, 2, 15 and 16 of a 28-day cycle. Of 33 carfilzomib-naive patients treated in the 20/27 mg m^−2^ (Cohort A) in this study 42% achieved a partial response or better. All of these patients were refractory or intolerant to bortezomib, and 27 of them were refractory or intolerant to lenalidomide. In contrast, none of the carfilzomib-refractory patients responded. Response data are not available for Cohort B, but the regimen appears to be well tolerated and demonstrates that carfilzomib dose can be escalated up to 56 mg m^−^^2^.^38^

### Side effects

The most common treatment-related AEs in both cohorts from the single-agent study described above included thrombocytopenia, anemia, neutropenia and fatigue. No treatment-emergent neuropathy was observed. The AEs had little effect on treatment continuation.^37^ In combination with carfilzomib, no major added toxicity was seen.^38^

### Dosing schedule

The recommended dosing schedule for single-agent therapy with filanesib is 1.5 mg m^−^^2^ administered intravenously on days 1, 2, 15 and 16 of a 28-day cycle. ^37^

No dose modification was required when combining the drug with carfilzomib.

### Ongoing trials

A multicenter international phase 2 trial (AFFIRM) of single-agent filanesib in 160 patients with RRMM who have been previously treated with bortezomib and lenalidomide and have disease refractory to carfilzomib and/or pomalidomide is currently ongoing.

Studies are also ongoing in combination with bortezomib and dexamethasone, and with pomalidomide–dexamethasone in the same patient population.

### Anticipated Role in MM

As with dinaciclib, this drug also has significant promise owing to its unique mechanism of action. Filanesib offers an alternative way to target the cell cycle, and differs from the currently available microtubule-targeting drugs.^33^ On the basis of the results of clinical studies thus far it has significant antimyeloma activity and efficacy, alone and in combination with other myeloma drugs. It has the potential to become a valuable component of the antimyeloma chemotherapeutic arsenal. It may also be possible to identify patients who will be more responsive to filanesib treatment by measuring serum levels of a protein called alpha 1-acid glycoprotein. Low levels of alpha 1-acid glycoprotein have been shown to predict a better response to filanesib and therefore offer an efficient way to screen candidates for both study and treatment.^39^

## Venetoclax (formerly referred to as ABT-199*;* AbbVie; Abbott and Genentech, North Chicago, IL, USA)

### Mechanism of action

Venetoclax (ABT-199) is a potent, selective oral drug that specifically acts to inhibit Bcl-2, a well-known regulator of apoptosis. Myeloma cells are especially sensitive to drugs that target pro-survival proteins such as Bcl-2. Venetoclax acts as a direct inhibitor of Bcl-2 and has shown significant activity in a variety of Bcl2-dependent hematologic malignancies. Venetoclax induces cell death in MM cell lines both *in vitro* and in primary MM samples *ex vivo*. It has been found that particular subtypes are more sensitive to venetoclax than others. MM cells with the *t*(11;14) translocation tend to express a higher ratio of Bcl-2 to Mcl-1 (Mcl1 being a resistance factor).^40^ Other preclinical studies thus far have shown that venetoclax administered in combination with melphalan or carfilzomib produces additive effects in a number of cell lines, especially when given with dexamethasone. The significant increase in cell death observed when venetoclax is combined with dexamethasone is thought to be because of an increase in the expression of both Bcl-2 and Bim (another pro-survival protein) upon the addition of dexamethasone. By altering the binding of Bim to anti-apopototic proteins, dexamethasone can induce greater affinity of Bim toward Bcl-2, resulting in increased sensitivity to venetoclax.^41^ Similarly, venetoclax may enhance the activity of bortezomib; this has been observed in MM xenograft models and is probably related to the inhibitory effect of bortezomib on Mcl-1 activity.^42, 43^ Given these interactions, additional drug combinations may be discovered, and warrant further testing and clinical trials.

### Clinical results in myeloma

A phase 1 single-agent trial of Venetoclax studied in 37 RRMM patients who had a median of 6 (range 1–19) prior lines of therapy was carried out to assess and determine a recommended phase 2 dosage, safety and efficacy profile, and the impact of chromosomal abnormalities on response. Of these 37 patients, 32 had prior bortezomib, 35 had lenalidomide treatment and 26 had prior stem cell transplant. Fourteen patients had *t*(11;14), 4 had t(4:14), 5 had del 17p and 17 had del 13q. Venetoclax was administered orally daily at 300, 600, 900 or 1200 mg after a 2-week dose ramp-up in the dose-escalation cohorts, and 1200 mg daily after ramp-up in the safety expansion cohort. Preliminary results of this phase 1 study show that, of the 32 evaluable patients, 6%(2/32) both with *t*(11;14) achieved a complete response (1 at 600 and the other at 900 mg). Of the 16 patients on the 1200-mg dosage in both cohorts (6 of 16 patients, 38% had *t*(11;14)), 6 experienced progressive disease, 5 achieved stable disease and 5 are not yet evaluable. An overall response rate of 15% was noted in the 13 patients with *t*(11;14) versus 0% in the 19 patients without *t*(11;14). These early results suggest that venetoclax has single-agent activity, most prominently in *t*(11;14) patients.^40^

Another phase 1b trial was initiated to determine the safety and efficacy of venetoclax in combination with bortezomib and dexamethasone in RRMM patients. Venetoclax was administered in 50–800 mg oral doses daily in combination for cycles 1–11 and alone in cycles 12 and beyond. Bortezomib 1.3 mg m^−^^2^ was given subcutaneously on days 1, 4, 8 and 11, and dexamethasone 20 mg PO on days 1, 2, 4, 5, 8, 9, 11 and 12 of a 21-day cycle for eight cycles. During cycles 9–11, bortezomib and dexamethasone were administered once weekly (days 1, 8, 15 and 22) of a 35-day cycle. The median number of prior lines of therapy was 5 (range 1–15). Of these patients, 35 were refractory to prior bortezomib treatment, 34 to prior lenalidomide and 29 to stem cell transplantation. Of the 40 evaluable patients, responses were observed only in bortezomib-sensitive or -naive patients. An overall response rate (partial response or better) was observed in 20/40 (50%) patients. The study is ongoing and enrolling patients in the 1000-mg dose-escalation cohort.^43^

### Side effects

The most common AEs observed (in >20% of patients) have been nausea (49%), anemia (27%) and fatigue (24%). Other hematologic drug-related AEs include thrombocytopenia (22%) and neutropenia (11%). The only serious AEs occurring infrequently included pyrexia, cough and sepsis, possibly related to venetoclax.^40^ Venetoclax is relatively well tolerated in trials where it is used in combination with other drugs.

### Dosing schedules

The recommended MTD and schedule for single-agent therapy with Venetoclax is currently 1200 mg administered orally on a daily basis after a 2-week dose ramp-up. This dosing schedule is being evaluated in a safety expansion cohort of a phase 1 trial.^40^

### Ongoing trials

Venetoclax was granted priority review in January 2016 by the Food and Drug Administration for use in the treatment of patients with CLL, and was recently approved for use in CLL. Venetoclax is currently under investigation in various clinical trials to determine its role and efficacy in myeloma and other hematologic malignancies (www.clinicaltrials.gov). Ongoing phase 2 and 3 trials are evaluating the role of the drug as a single agent and in various combinations.

### Anticipated role in myeloma

Venetoclax can potentially have a major role in the treatment of MM and also of various other hematological malignancies. In fact, it has recently been approved for the treatment of a subset of patients with chronic lymphocytic leukemia. Its activity as a single agent in myeloma therapy is promising, especially in *t*(11;14) myeloma, as seen in the ongoing clinical trials described above. With its unique mechanism of action and evident ability thus far to enhance the activity of drugs, it is administered in combination, and is beneficial for future use in myeloma therapy as well as for manufacturing new chemotherapeutic multidrug regimens. It has also exhibited minimal side effects and is well tolerated, and will enable the development of new non-cross-resistant regimens.

## LGH447 (Novartis Pharmaceuticals, Basel, Switzerland)

### Mechanism of action

The final new drug in the arsenal of myeloma drugs that is being sought after and tested is still unnamed, and is simply known as LGH447. It is a pan PIM kinase inhibitor. Pims make up a group of serine/threonine kinases that act to regulate and express cell cycle progression and survival.^44^ Elevated levels of Pim1 and 2 have been found across all hematological malignancies, specifically in MM with the highest level of Pim-2 expression, making it an ideal target for anticancer therapy. Pim kinases can affect cytokines and growth factor signaling pathways downstream as well, therefore having an indirect role in cell cycle progression and inhibition of apoptosis in many hematologic malignancies. They are felt to mediate myeloma cell proliferation and survival while also having a role in the frequent bone destruction seen in this disease. Pim kinases also typically act to promote oncogenic signaling in the hypoxic bone marrow environment, allowing resistance to therapy to develop. LGH447 is an oral drug whose mechanism of action is via inhibition of pan-Pim protein kinases (Pim-1, Pim-2 and Pim-3). By interfering with the interactions and activities of Pim kinases, LGH447 can interrupt crucial phases (G1/S) in the cell cycle and affect expression of various pro-apoptotic proteins such as Bcl-2.

LGH447 has been tested in MM and AML models and has shown significant activity as a pan-PIM kinase inhibitor according to a study by Garcia *et al.*^45^ At first, mutations were identified in murine T-cell lines showing expression of PIM1, 2 and 3 kinases belonging to the larger PIM kinase family.^46^ Preclinical studies showed evidence that LGH447 was active in PIM2-dependent MM cell lines by inhibiting proliferation, mTOR-C1 signaling and inhibiting tumor growth in mouse and subcutaneous xenograft models. LGH447 was also observed to have appreciably reduce the bone tumor burden in a orthotopic human xenograft model of MM, thereby necessitating the need for further clinical trials in patients with MM both as a single agent and in combination therapy.

### Clinical results in myeloma

The first in-human, multicenter open-label, phase 1 dose-escalation study on LGH447 was conducted by Raab *et al.*^47^ in 54 patients with RRMM, for whom no other effective treatment options were available. LGH447 was administered orally once daily starting at 70 mg (*n=*5), 150 mg (*n=*6), 200 mg (*n=*6), 250 mg (*n=*7), 300 (*n=*4), 350 mg (*n=*10), 500 mg (*n=*10) and 700 mg (*n=*6), with the MTD determined to be 500 mg once daily.^47^ All patients had a history of prior treatment with a median of at least 4 (range 1–6) lines of therapy. At least 81% had received a proteasome inhibitor, 83% prior immunomodulatory therapy, 70.4% lenalidomide, 48% thalidomide, 68% received both a proteasome inhibitor and IMiDs, and 87% prior stem cell treatment. Of the 48 evaluable patients, single-agent activity was most evident at doses >150 mg; five patients (11%) had partial responses or better at doses ranging from 150 to 500 mg (one of whom had a very good partial response at 200 mg). Minimal response was observed in 5 of the 48 patients. Pim kinase inhibition therefore has a promising therapeutic role in MM patients and should be further studied clinically.

### Side effects

The most common AEs were thrombocytopenia (19%), anemia (19%), neutropenia (13%) and fatigue (11%), and the only reported unexpected serious AE was vasovagal syncope and was seen in one patient in an isolated episode.^47^

### Dosing schedules

There is no currently recommended dosing schedule available, as phase 1b/2 clinical trials are still underway. However, preliminary results suggest that a dose of 500 mg once daily oral is likely the MTD of LGH 447.

### Anticipated role in myeloma

A study by Lu *et al.*^48^ uncovered a key concept fundamental to the proliferation and maintenance of cell growth in MM. Pim2 expression, as stated early, was found to be highly elevated in MM and a requirement to promote the activity of cell growth. As LGH447 can directly target Pim-2 molecules found in high expression in MM cells, there is a significant therapeutic rationale worth pursuing and studying further. The effect of blocking Pim2 activity can inhibit proliferation more than increasing apoptosis.^48^ The anti-apoptotic function of Pim kinases is likely to be observed when they are in the presence of death-promoting stimuli. Therefore, Pim2 inhibitors may enhance the effect of apoptosis-promoting agents like bortezomib and thalidomide. LGH447 may well be an important component of current myeloma therapy that along with other active agents ultimately helps shut down myeloma cell proliferation, especially in patients with RRMM.

## Summary

There has been remarkable progress in the identification of new active agents in MM. Each of the drugs discussed in this review are ones with meaningful single-agent activity and have a high likelihood of being approved for clinical use once appropriate regulatory studies are conducted. As many of these possess new mechanisms of action, we can develop numerous active multidrug combinations, as step essential for the development of a curative strategy. Having new agents approved in the near future that can act as antimyeloma agents in various ways can allow us to discover new combinations for approach to treatment and can ultimately provide us with long-term success.^49^

Major advances in the diagnosis and treatment of MM have occurred in the last decade. Future trials should address the optimal sequencing of the various treatment regimens available, the incorporation of monoclonal antibodies to existing regimens in a cost-effective and safe manner, the role of MRD as a goal of therapy, optimal treatment of high-risk MM and extramedullary disease, and early intervention toward a cure of the disease. With so many active agents, we do face a daunting task of designing phase 3 studies that can inform the best sequence of therapy, the most effective combinations and the optimal strategy by which we can maximize efficacy with the lowest amount of side effects and cost.

## Figures and Tables

**Table 1:** Investigational drugs with significant single-agent activity in multiple myeloma

*Agent*	*Dosing schedule used in clinical trials*	*Postulated mechanism of action*	*Side effects*
Isatuximab^20^	20 mg kg^−1^ weekly × 4, followed by maintenance therapy of 10 mg kg^−1^ IV Q2W	Monoclonal antibody targeting CD38	Infusion-related reactions, anemia, thrombocytopenia, fatigue, nausea
Marizomib^24^	0.5 mg m^−^^2^ IV over 2 h, Days 1, 4, 8 and 11 of a 21-day cycle	Inhibits the ubiquitin–proteasome catalytic pathway in cells by binding directly with the 20S proteasome complex	Dizziness, fever, fatigue, nausea, hallucinations
Oprozomib^26^	240 mg per day once daily, Days 1, 2, 8 and 9 of a 14-day cycle (+30 mg increments as tolerated to max 300 mg per day) OR 150 mg per day once daily Days 1–5 of a 14-day cycle (+30 mg increments as tolerated to max 180 mg per day)	Inhibits the ubiquitin–proteasome catalytic pathway in cells by binding directly with the 20S proteasome complex	Hypotension, thrombocytopenia, anemia, diarrhea, vomiting, nausea
Venetoclax^40^	1200 mg PO once daily (after a 2-week dose ramp-up)	Inhibits Bcl-2, a well-known regulator of apoptosis	Neutropenia, thrombocytopenia, anemia, fatigue, nausea
Dinaciclib^30^	50 mg m^−2^ IV infusion over 2 h, Day 1 of a 21-day cycle for a maximum of 12 cycles	Inhibits CDK in the cell cycle	Leukopenia, thrombocytopenia, fatigue, gastrointestinal side effects
Filanesib^38^	1.5 mg m^−^^2^ IV, Days 1, 2, 15 and 16 of a 28-day cycle	Inhibits KSP and thereby cell division	Neutropenia, thrombocytopenia, anemia, fatigue
LGH447^(ref. 47)^	500 mg PO once daily	Inhibits PIM kinase, which acts to regulate and express cell cycle progression and survival	Neutropenia, thrombocytopenia, anemia

Abbreviations: CDK, cyclin-dependent kinase; IV, intravenous; KSP, kinesin spindle protein; PIM kinase, proto-oncogene serine/threonine-protein kinase; PO, per ora.

**Table 2:** Reported single-agent activity of new drugs in relapsed refractory multiple myeloma

*Agent*	*Patient characteristics*	*No. of patients*^a^	*PRR (%)*	*ORR (partial+minor)*
Isatuximab^20^	Median five prior lines of therapy (range 2–10). 88% Dual refractory^b^	25 (Treated at MTD)	24	24%
Marizomib^24^	Median five to seven prior lines of therapy	68	0	3%
Oprozomib^26^	Bortezomib refractory	12	25	NR
	Carfilzomib refractory	11	27	NR
Venetoclax^40^	Median six prior therapies (range 1–19), >85% previously treated with lenalidomide and bortezomib. *t*(11;14) seen in 13 patients versus without *t*(11;14) in 19 patients	32	15 0	NR NR
Dinaciclib^30^	Up to five prior lines of therapy	27	11	48%
Filanesib^38^	Median six prior lines of therapy (range 2–19)	32	16	19%
LGH447^(ref. 47)^	Median four prior lines of therapy (range 1–6)	48	11	21%

Abbreviations: MTD, maximum tolerated dose; NR, not reported; ORR, overall response rate; PRR, partial response rate.

a Reflects only evaluable patients in study for the cohort of interest.

b Dual Refractory, indicates refractory to lenalidomide and bortezomib.

**Table 3:** Comparison of proteasome inhibitors

*Feature*	*Carfilzomib (Kyprolis)*	*Bortezomib (Velcade)*	*Ixazomib (Ninlaro)*	*Marizomib (NPI-0052)*	*Oprozomib (ONX-0912)*
Nature of inhibition	Irreversible inhibition	Reversible inhibition	Reversible inhibition	Irreversible inhibition	Irreversible inhibition
Chemical structure	Tetrapeptide epoxyketone	Boronic acid	Boronic acid derivative	Salinosporamide	Tripeptide epoxyketone
specificity	Highly selective for chymotrypsin-like active site, β-5	Inhibits both chymotrypsin-like and caspase-like sites, β-5 and β-1	Preferentially inhibits chymotrypsin-like site, β-5	Inhibits all three subunits of the proteasome, β-5, β-1 and β-2	Highly selective for chymotrypsin-like active site, β-5
Route of administration	Intravenous	Intravenous or subcutaneous	Oral	Intravenous	Oral
Side effects/neuropathy risk	Minimal peripheral neuropathy	Risk of peripheral neuropathy	Mild neuropathy and gastrointestinal side effects	Peripheral neuropathy uncommon	Gastrointestinal side effects, no neuropathy
Recommended dosing schedule	20/27 mg m^−^^2^ On days 1, 2, 8, 9, 15 and 16 every 28 days	0.7–1.3 mg m^−^^2^ Once weekly days 1, 8, 15 and 22 every 28 days	4 mg Once weekly on days 1, 8 and 15 every 28 days	0.5 mg m^−^^2^ Over 120 min days 1, 4, 8 and 11 of a 21-day cycle	240/300 mg Per day twice a week, or 150/180 mg per day, 5 days in 2 weeks
US Food and Drug Administration approval status	Approved	Approved	Approved	Not approved	Not approved
